# Prediction of Survival Among Patients Receiving Transarterial Chemoembolization for Hepatocellular Carcinoma: A Response‐Based Approach

**DOI:** 10.1002/hep.31022

**Published:** 2020-05-27

**Authors:** Guohong Han, Sarah Berhane, Hidenori Toyoda, Dominik Bettinger, Omar Elshaarawy, Anthony W. H. Chan, Martha Kirstein, Cristina Mosconi, Florian Hucke, Daniel Palmer, David J. Pinato, Rohini Sharma, Diego Ottaviani, Jeong W. Jang, Tim A. Labeur, Otto M. van Delden, Mario Pirisi, Nick Stern, Bruno Sangro, Tim Meyer, Waleed Fateen, Marta García‐Fiñana, Asmaa Gomaa, Imam Waked, Eman Rewisha, Guru P. Aithal, Simon Travis, Masatoshi Kudo, Alessandro Cucchetti, Markus Peck‐Radosavljevic, R.B. Takkenberg, Stephen L. Chan, Arndt Vogel, Philip J. Johnson

**Affiliations:** ^1^ Department of Liver Disease and Digestive Interventional Radiology Xijing Hospital of Digestive Disease Fourth Military Medical University Xi’an China; ^2^ Department of Biostatistics University of Liverpool Liverpool United Kingdom; ^3^ Department of Gastroenterology and Hepatology Ogaki Municipal Hospital Ogaki Japan; ^4^ Department of Medicine II Faculty of Medicine Medical Center University of Freiburg University of Freiburg Freiburg Germany; ^5^ National Liver Institute Menoufia University Shebeen El‐Kom Egypt; ^6^ Department of Pathology Chinese University of Hong Kong Hong Kong; ^7^ Department of Gastroenterology, Hepatology and Endocrinology Hannover Medical School Hannover Germany; ^8^ Radiology Unit Department of Specialized Diagnostic and Experimental Medicine Alma Mater Studiorum ‐ University of Bologna Italy University Hospital of Bologna Sant'Orsola‐Malpighi Polyclinic Bologna Italy; ^9^ Department of Internal Medicine and Gastroenterology Klinikum Klagenfurt am Wörthersee Klagenfurt Austria; ^10^ Department of Molecular and Clinical Cancer Medicine University of Liverpool Liverpool United Kingdom; ^11^ Department of Surgery and Cancer Imperial College London London United Kingdom; ^12^ UCL Cancer Institute University College London London United Kingdom; ^13^ Department of Internal Medicine The Catholic University of Korea Seoul St. Mary’s Hospital Seoul Republic of Korea; ^14^ Department of Gastroenterology and Hepatology Amsterdam University Medical Center Amsterdam the Netherlands; ^15^ Department of Radiology Amsterdam University Medical Centers Amsterdam the Netherlands; ^16^ Department of Translational Medicine Università del Piemonte Orientale Novara Italy; ^17^ Department of Gastroenterology and Hepatology Aintree University Hospital Liverpool United Kingdom; ^18^ Liver Unit Clínica Universidad de Navarra IDISNA and CIBEREHD Pamplona Spain; ^19^ Research Department of Oncology UCL Cancer Institute University College London London United Kingdom; ^20^ National Institute for Health Research Nottingham Biomedical Research Centre Nottingham University Hospitals National Health Service Trust and the University of Nottingham Nottingham United Kingdom; ^21^ Nottingham Digestive Diseases Centre School of Medicine University of Nottingham Nottingham United Kingdom; ^22^ Department of Radiology Nottingham University Hospitals National Health Service Trust Nottingham United Kingdom; ^23^ Department of Gastroenterology and Hepatology Kinki University School of Medicine Osaka‐Sayama Osaka Japan; ^24^ Department of Medical and Surgical Sciences University of Bologna Bologna Italy; ^25^ Department of Clinical Oncology Chinese University of Hong Kong Shatin Hong Kong

## Abstract

**Background and Aims:**

The heterogeneity of intermediate‐stage hepatocellular carcinoma (HCC) and the widespread use of transarterial chemoembolization (TACE) outside recommended guidelines have encouraged the development of scoring systems that predict patient survival. The aim of this study was to build and validate statistical models that offer individualized patient survival prediction using response to TACE as a variable.

**Approach and Results:**

Clinically relevant baseline parameters were collected for 4,621 patients with HCC treated with TACE at 19 centers in 11 countries. In some of the centers, radiological responses (as assessed by modified Response Evaluation Criteria in Solid Tumors [mRECIST]) were also accrued. The data set was divided into a training set, an internal validation set, and two external validation sets. A pre‐TACE model (“Pre‐TACE‐Predict”) and a post‐TACE model (“Post‐TACE‐Predict”) that included response were built. The performance of the models in predicting overall survival (OS) was compared with existing ones. The median OS was 19.9 months. The factors influencing survival were tumor number and size, alpha‐fetoprotein, albumin, bilirubin, vascular invasion, cause, and response as assessed by mRECIST. The proposed models showed superior predictive accuracy compared with existing models (the hepatoma arterial embolization prognostic score and its various modifications) and allowed for patient stratification into four distinct risk categories whose median OS ranged from 7 months to more than 4 years.

**Conclusions:**

A TACE‐specific and extensively validated model based on routinely available clinical features and response after first TACE permitted patient‐level prognostication.

**Abbreviations**
AFP: alpha‐fetoprotein
ALBI: albumin‐bilirubin
BCLC: Barcelona Clinic Liver Cancer
CI: confidence interval
CR: complete response
DAA: direct‐acting antiviral
DEB: drug‐eluting bead
HAP: hepatoma arterial embolization prognostic
HBV: hepatitis B virus
HCC: hepatocellular carcinoma
HCV: hepatitis C virus
HR: hazard ration
KM: Kaplan‐Meier
mHAP‐II: modified HAP‐II
mHAP‐III: modified HAP‐III
mRECIST: modified Response Evaluation Criteria in Solid Tumors
NAFLD: nonalcoholic fatty liver disease
OS: overall survival
PD: progressive disease
PR: partial response
SD: stable disease
SVR: sustained virological response
TACE: transarterial chemoembolization
VI: vascular invasion

International guidelines recommend transarterial chemoembolization (TACE) for patients with hepatocellular carcinoma (HCC) at the Barcelona Clinic Liver Cancer (BCLC) intermediate stage (B) or for those at the BCLC 0/A stage who are not candidates for percutaneous ablation, liver resection, or transplantation by virtue of the tumor location, portal hypertension, or comorbidity.^1^, ^2^ This recommendation was based on two randomized trials and subsequent studies.^3^, ^4^, ^5^, ^6^, ^7^ However, the heterogeneity of this “intermediate” population has been extensively documented, and the unmet need of stratification according to baseline features has been emphasized.^8^, ^9^

Among those in the cohort who are classified as “ideal candidates” for TACE, an expected median survival in the order of 30 months is quoted, but even within this patient group, there is a wide variation in survival.^5^, ^6^, ^10^ However, in practice, many patients receive TACE outside the guideline criteria. For example, vascular invasion (VI) is not always considered a contraindication to TACE^11^; therefore, in this expanded population, variation in survival may be even greater. This wide variability in survival has led to attempts to define the prognostic features and combine these into scores (or “models”) that can be applied to assess prognosis at a subgroup or individual patient level. One frequently quoted aim is to identify that subgroup of patients who respond poorly to TACE and may be considered for systemic therapies.^8^, ^12^

Among the first prognostic scores to be developed was the hepatoma arterial embolization prognostic (HAP) score, which is based on a simple points system involving tumor size, alpha‐fetoprotein (AFP), bilirubin, and albumin.^13^ The HAP score (which was enhanced by Kim et al.^14^ by adding tumor number [referred to as the modified HAP‐II {mHAP‐II}]) has the advantage of easy applicability and simplicity but does not permit individual patient‐level prognostication. This limitation was overcome by Cappelli et al., who developed the modified HAP‐III (mHAP‐III) to include HAP variables, together with tumor number in their continuous (as opposed to dichotomized) form.^15^ mHAP‐III permits individual patient‐level prognostication expressed as the likelihood of survival at a specific period of time after the first TACE.

A second, and more important, limitation of current scores is that they may be HCC‐specific rather than TACE‐specific.

In this study, it was confirmed that the HAP score is HCC‐specific rather than TACE‐specific, and we present TACE‐specific models that permit accurate individualized patient survival prediction.

## Patients and Methods

This analysis was reported according to the Transparent Reporting of a Multivariable Prediction Model for Individual Prognosis or Diagnosis guidelines.^16^

As a prelude to the main study, the specificity of the HAP score for patients undergoing TACE was examined in 3,556 patients with early HCC who underwent resection and in 967 patients with advanced HCC who received sorafenib within clinical trials.^17^, ^18^

In the main study, the reported TACE cohort^19^ was expanded by collecting further cases in which the response to TACE according to the modified Response Evaluation Criteria in Solid Tumors (mRECIST)^20^, ^21^ was recorded. This analysis has involved only patients who were classified by the local investigator as undergoing TACE as their primary and first treatment. Patients whose TACE was used as a bridge to transplantation or other potentially curative treatment options were excluded, as were patients with extrahepatic metastasis. The study protocol conformed to the ethical guidelines of the 1975 Declaration of Helsinki as reflected in a priori approval by the appropriate institutional review committee.

All participating centers had specific expertise in the management of HCC and the practice of TACE. There were 19 centers representing 11 different countries, including a reported multicenter cohort^22^, ^23^ that comprised patients from London (United Kingdom), Osaka (Japan), Seoul (Korea), and Novara (Italy) (Tables 1 and 2). Most centers used “conventional” TACE, although several moved to drug‐eluting bead (DEB)–based TACE after 2008. In all centers, patients were followed up by computed tomography (CT) or magnetic resonance imaging scans once every 3 months after stable disease (SD) had been attained.

**Table 1:** Patient Characteristics

Variable	Xi’an, China (N = 786)	Freiburg, Germany (N = 407)	Menofia, Egypt (N = 391)	Hannover, Germany (N = 356)	Hong Kong 1 (N = 140)	Hong Kong 2 (N = 242)	Bologna, Italy (N = 234)	Ogaki, Japan (N = 613)	Amsterdam, NL (N = 138)	Pamplona, Spain (N = 85)	Birmingham, UK (N = 167)	Liverpool, UK (N = 132)	London, UK 1 (N = 114)	London, UK 2 (N = 84)	Nottingham, UK (N = 41)	Klagenfurt, Austria (N = 220)	Multicenter* (N = 471)
Age (years)	54 (11.9), n = 785	67 (9.3), n = 407	59 (8.3), n = 391	64 (11.0), n = 356	64 (10.4), n = 140	62 (11.3), n = 242	65 (9.7), n = 234	65 (9.7), n = 613	68 (9.8), n = 138	64 (10.5), n = 84	64 (10.3), n = 166	69 (9.4), n = 132	64 (10.1), n = 114	65 (9.6), n = 84	70 (8.8), n = 41	67 (9.8), n = 220	69 (10.6), n = 471
Male, n (%)	654 (83.9), n = 780	349 (85.8), n = 407	282 (72.1), n = 391	286 (80.3), n = 356	121 (86.4), n = 140	209 (86.4), n = 242	177 (75.6), n = 234	456 (74.4), n = 613	106 (76.8), n = 138	72 (84.7), n = 85	133 (79.6), n = 167	112 (84.9), n = 132	99 (86.8), n = 114	73 (86.9), n = 84	33 (80.5), n = 41	189 (85.9), n = 220	348 (73.9), n = 471
Cause, n (%)	n = 786	n = 407	n = 379	n = 354	n = 140	n = 242	n = 233	n = 610	n = 133	n = 81	n = 94	n = 121	n = 106	n = 83	n = 41	n = 205	n = 471
HCV	19 (2.4)	87 (21.4)	347 (91.6)	82 (23.2)	11 (7.9)	18 (7.4)	129 (55.4)	349 (57.2)	29 (21.8)	42 (51.9)	26 (27.7)	10 (8.3)	27 (25.5)	23 (27.7)	5 (12.2)	63 (30.7)	232 (49.3)
HBV	708 (90.1)	42 (10.3)	24 (6.3)	56 (15.8)	111 (79.3)	196 (81.0)	27 (11.6)	108 (17.7)	11 (8.3)	9 (11.1)	16 (17.0)	2 (1.7)	17 (16.0)	8 (9.6)	0 (0)	16 (7.8)	98 (20.8)
Alcohol	1 (0.1)	154 (37.8)	0 (0)	100 (28.3)	0 (0)	0 (0)	27 (11.6)	0 (0)	43 (32.3)	15 (18.5)	42 (44.7)	32 (26.5)	16 (15.1)	10 (12.1)	14 (34.2)	102 (49.8)	85 (18.1)
ther	58 (7.4)	124 (30.5)	8 (2.1)	116 (32.8)	18 (12.9)	28 (11.6)	50 (21.5)	153 (25.1)	50 (37.6)	15 (18.5)	10 (10.6)	77 (63.6)	46 (43.4)	42 (50.6)	22 (53.7)	24 (11.7)	56 (11.9)
ECOG 0/1, n (%)	n = 786	n = 407	n = 391	N/A	N/A	n = 125	n = 234	N/A	n = 132	n = 85	n = 40	N/A	n = 57	n = 74	n = 41	n = 220	N/A
0	427 (54.3)	311 (76.4)	324 (82.9)	N/A	N/A	55 (44.0)	192 (82.1)	N/A	62 (47.0)	72 (84.7)	26 (65.0)	N/A	35 (61.4)	40 (54.1)	24 (58.5)	220 (100)	N/A
1	355 (45.2)	46 (11.3)	67 (17.1)	N/A	N/A	68 (54.4)	42 (18.0)	N/A	54 (40.9)	10 (11.8)	9 (22.5)	N/A	13 (22.8)	22 (29.7)	12 (29.3)	0 (0)	N/A
2	4 (0.5)	50 (12.3)	0 (0)	N/A	N/A	1 (0.8)	0 (0)	N/A	15 (11.4)	2 (2.4)	3 (7.5)	N/A	9 (15.8)	11 (14.9)	5 (12.2)	0 (0)	N/A
3	0 (0)	0 (0)	0 (0)	N/A	N/A	1 (0.8)	0 (0)	N/A	1 (0.8)	1 (1.2)	2 (5.0)	N/A	0 (0)	1 (1.4)	0 (0)	0 (0)	N/A
Baseline Child‐Pugh grade, n (%)	n = 786	n = 407	n = 391	n = 338	n = 140	n = 242	n = 234	n = 613	n = 134	n = 85	n = 167	n = 132	n = 91	n = 83	n = 40	n = 220	n = 469
A	712 (90.6)	291 (71.5)	283 (72.4)	230 (68.1)	107 (76.4)	195 (80.6)	156 (66.7)	320 (52.2)	104 (77.6)	51 (60.0)	151 (90.4)	120 (90.9)	68 (74.7)	70 (84.3)	27 (67.5)	136 (61.8)	343 (73.1)
B	72 (9.2)	104 (25.6)	108 (27.6)	105 (31.1)	31 (22.1)	43 (17.8)	71 (30.3)	255 (41.6)	29 (21.6)	31 (36.5)	16 (9.6)	12 (9.1)	22 (24.2)	13 (15.7)	11 (27.5)	84 (38.2)	124 (26.4)
C	2 (0.3)	12 (3.0)	0 (0)	3 (0.9)	2 (1.4)	4 (1.7)	7 (3.0)	38 (6.2)	1 (0.8)	3 (3.5)	0 (0)	0 (0)	1 (1.1)	0 (0)	2 (5.0)	0 (0)	2 (0.4)
Median follow‐up, months (95% CI)	45.0 (41.7, 51.2), n = 784	89.2 (68.4, 129.0), n = 406	47.3 (44.7, 50.9), n = 3,420														
Median OS, months (95% CI)	14.6 (13.0, 16.6), n = 784	17.6 (14.8, 20.4), n = 406	21.2 (20.3, 22.2), n = 3,420														

* Centers involved London (UK), Osaka (Japan), Seoul (Korea), and Novara (Italy).

Abbreviations: ECOG, Eastern Cooperative Oncology Group; N/A, not applicable; NL, the Netherlands; UK, United Kingdom.

**Table 2:** Tumor Characteristics and Laboratory Results

Variable	Xi’an, China (N = 786)	Freiburg, Germany (N = 407)	Menofia, Egypt (N = 391)	Hannover, Germany (N = 356)	Hong Kong 1 (N = 140)	Hong Kong 2 (N = 242)	Bologna, Italy (N = 234)	Ogaki, Japan (N = 613)	Amsterdam, NL (N = 138)	Pamplona, Spain (N = 85)	Birmingham, UK (N = 167)	Liverpool, UK (N = 132)	London, UK 1 (N = 114)	London, UK 2 (N = 84)	Nottingham, UK (N = 41)	Klagenfurt, Austria (N = 220)	Multicenter* (N = 471)
Solitary tumors, n (%)	396 (51.2), n = 774	132 (32.5), n = 406	161 (41.2), n = 391	77 (21.8), n = 353	59 (42.5), n = 139	82 (33.9), n = 242	108 (46.2), n = 234	190 (31.1), n = 612	42 (30.4), n = 138	27 (31.8), n = 85	59 (36.7), n = 161	63 (47.7), n = 132	48 (42.5), n = 113	30 (35.7), n = 84	18 (43.9), n = 41	73 (33.2), n = 220	107 (27.3), n = 392
Tumor size (cm)	8.5 (5.5, 11.8), n = 741	5.0 (3.2, 7.6), n = 407	4.5 (3.4, 5.9), n = 391	4.8 (3.1, 7.6), n = 329	5.9 (3.8, 10), n = 136	6.3 (4, 10), n = 230	3 (1.9, 4.3), n = 234	3.4 (2.2, 5.1), n = 564	5.0 (3.9, 6.8), n = 137	6 (3.3, 9.0), n = 79	5.1 (4.0, 7.9), n = 154	4.6 (3.3, 6.8), n = 132	5.0 (3.2, 7.3), n = 109	3.8 (2.1, 6.4), n = 84	5.0 (3.5, 10.7), n = 41	4.0 (3.0, 6.3), n = 220	3.5 (2.2, 5.8), n = 471
VI, n (%)	242 (30.8), n = 786	20 (4.9), n = 407	0 (0), n = 436	42 (11.9), n = 352	14 (10.0), n = 140	34 (14.1), n = 242	2 (0.9), n = 234	168 (27.5), n = 612	8 (5.8), n = 138	12 (14.1), n = 85	47 (28.1), n = 167	5 (3.8), n = 131	7 (6.2), n = 113	0 (0)	4 (9.8), n = 41	0 (0)	44 (9.3), n = 471
Baseline ALBI grade	n = 784	n = 407	n = 391	n = 355	n = 140	n = 242	n = 234	n = 612	n = 124	n = 75	n = 167	n = 132	n = 97	n = 82	n = 41	n = 220	n = 389
1	337 (43.0)	128 (31.5)	89 (22.8)	95 (26.8)	35 (25.0)	94 (38.8)	58 (24.8)	81 (13.2)	66 (53.2)	17 (22.7)	78 (46.7)	58 (43.9)	28 (28.9)	35 (42.7)	5 (12.2)	51 (23.2)	124 (31.9)
2	434 (55.4)	244 (60.0)	262 (67.0)	230 (64.8)	94 (67.1)	135 (55.8)	158 (67.5)	434 (70.9)	48 (38.7)	46 (61.3)	87 (52.1)	71 (53.8)	60 (61.9)	43 (52.4)	31 (75.6)	150 (68.2)	144 (37.0)
3	13 (1.7)	35 (8.6)	40 (10.2)	30 (8.5)	11 (7.9)	13 (5.4)	18 (7.7)	97 (15.9)	10 (8.1)	12 (16.0)	1 (1.2)	3 (2.3)	9 (9.3)	4 (4.9)	5 (12.2)	19 (8.6)	121 (31.1)
Baseline ALBI score	−2.50 (0.5), n = 784	−2.26 (0.6), n = 407	−2.15 (0.6), n = 391	−2.21 (0.6), n = 355	−2.22 (0.5), n = 140	−2.35 (0.5), n = 242	−2.21 (0.5), n = 234	−1.97 (0.6), n = 612	−2.46 (0.6), n = 124	−2.07 (0.6), n = 75	−2.48 (0.5), n = 167	−2.52 (0.5), n = 132	−2.24 (0.7), n = 97	−2.42 (0.5), n = 82	−2.01 (0.5), n = 41	−2.19 (0.5), n = 220	−1.98 (−3.08, −1.24), n = 389
Baseline AFP (ng/mL)	356.2 (14.2, 3650.5), n = 776	46.7 (6.7, 472.2), n = 366	79 (12.1, 49 7), n = 391	44 (7, 391), n = 323	89.5 (9, 1356.5), n = 140	126.5 (16, 2300), n = 242	15 (5, 58), n = 191	43 (12, 410), n = 579	28 (5.5, 305.5), n = 128	8.3 (4, 659.7), n = 81	60 (6, 1287), n = 163	10.5 (3, 157.5), n = 100	87.3 (7.1, 1206), n = 102	73.6 (7.5, 469), n = 79	32.5 (4, 546.5), n = 40	26.6 (6, 290.1), n = 219	31.5 (8, 236), n = 466
Baseline albumin (g/L)	39 (5.4), n = 784	36 (6.1), n = 407	35 (5.8), n = 391	35 (5.9), n = 355	35 (5.2), n = 140	37 (5.2), n = 242	37 (5.1), n = 234	33 (6.1), n = 612	38 (5.6), n = 127	35 (6.0), n = 76	38 (5.2), n = 167	39 (4.7), n = 132	37 (7.0), n = 106	38 (5.3), n = 83	33 (4.7), n = 41	36 (5.4), n = 220	32.7 (23.4, 44.8), n = 389
Baseline bilirubin (µmol/L)	16.7 (11.7, 22.6), n = 784	17.1 (12.0, 25.7), n = 407	18.8 (13.7, 25.7), n = 391	15 (10, 24), n = 356	14 (9, 22), n = 140	17 (11, 24), n = 242	21.6 (14.0, 36.9), n = 234	15.4 (11.1, 23.9), n = 612	16 (8, 26), n = 127	27.7 (15. 6, 42.5), n = 84	14 (9, 24), n = 167	14 (9.5, 23), n = 132	20 (14, 32), n = 97	17 (12, 25), n = 82	15 (10, 22), n = 41	21.6 (14.4, 32.3), n = 220	13.7 (10.3, 21), n = 471
Baseline AST (IU/L)	50 (35, 75.5), n = 784	65 (43, 101), n = 407	65 (46, 93), n = 391	N/A	N/A	N/A	N/A	N/A	53 (35, 92), n = 126	N/A	51 (35, 84), n = 167	N/A	N/A	68.5 (44, 107.5), n = 80	51.5 (37.5, 76), n = 20	52 (34.5, 80), n = 220	53 (36, 75), n = 449
Baseline platelets (× 10^9^)	128 (81, 185), n = 786	155 (108, 221), n = 407	N/A	N/A	155 (91, 240), n = 138	162 (111, 252), n = 125	N/A	102 (69, 147), n = 500	142 (106, 195), n = 126	110 (76, 165), n = 85	N/A	N/A	N/A	130 (82, 202), n = 83	154 (110.5, 231.5), n = 40	117 (82, 173.5), n = 220	124 (85, 178), n = 392
Baseline INR	1.1 (1.0, 1.2), n = 778	1.1 (1.0, 1.2), n = 407	1.2 (1.1, 1.3), n = 391	N/A	1.1 (1.1, 1.2), n = 140	0.9 (0.9, 1.0), n = 242	1.3 (1.1, 1.4), n = 234	N/A	1.1 (1.1, 1.2), n = 122	1.2 (1.0, 1.2), n = 77	1.1 (1.0, 1.2), n = 167	1.1 (1.0, 1.2), n = 132	1.2 (1.1, 1.4), n = 103	1.2 (1.1, 1.3), n = 83	1.0 (0.9, 1.1), n = 41	N/A	1.1 (1.1, 1.2), n = 350
Baseline creatinine	80 (68, 93), n = 781	79.6 (61.9, 93.7), n = 406	72.5 (61.9, 96.4), n = 391	N/A	83 (72.5, 98.5), n = 140	N/A	N/A	N/A	76 (64, 91), n = 127	79.6 (70.7, 93.7), n = 82	87 (76, 101), n = 167	84 (73, 98), n = 132	87 (74, 99), n = 106	N/A	73 (61, 82), n = 41	80.4 (68.1, 96.4), n = 220	N/A
Response after first TACE	n = 786	n = 407	n = 390	N/A	N/A	N/A	n = 234	N/A	n = 105	N/A	N/A	N/A	N/A	N/A	n = 39	n = 212	n = 461
CR	133 (16.9)	6 (1.5)	167 (42.8)	N/A	N/A	N/A	125 (53.4)	N/A	18 (17.1)	N/A	N/A	N/A	N/A	N/A	7 (18.0)	11 (5.2)	158 (34.3)
PR	203 (25.8)	57 (14.0)	150 (38.5)	N/A	N/A	N/A	96 (41.0)	N/A	54 (51.4)	N/A	N/A	N/A	N/A	N/A	9 (23.1)	68 (32.1)	110 (23.9)
SD	268 (34.1)	230 (56.5)	49 (12.6)	N/A	N/A	N/A	2 (0.9)	N/A	11 (10.5)	N/A	N/A	N/A	N/A	N/A	10 (25.6)	116 (54.7)	80 (17.4)
PD	182 (23.2)	114 (28.0)	24 (6.2)	N/A	N/A	N/A	11 (4.7)	N/A	22 (21.0)	N/A	N/A	N/A	N/A	N/A	13 (33.3)	17 (8.0)	113 (24.5)

* Centers involved London (UK), Osaka (Japan), Seoul (Korea), and Novara (Italy).

Abbreviations: AST, aspartate transaminase; ECOG, Eastern Cooperative Oncology Group; N/A, not applicable; NL, the Netherlands; UK, United Kingdom.

Baseline variables available in all the centers were age, sex, cause (hepatitis C virus [HCV], hepatitis B virus [HBV], alcohol, or “other”), tumor number (solitary or multiple), tumor size (centimeters), VI, Child‐Pugh grade, albumin (grams per liter), bilirubin (micromoles per liter), and AFP (nanograms per milliliter). The approach to TACE (DEB‐based or lipiodol‐based methods) was not proscribed, although no case received transarterial radioembolization.

The “other” cause comprised mainly patients with nonalcoholic fatty liver disease (NAFLD), other types of chronic liver disease, and more than one cause. The first TACE procedure was undertaken within 6 weeks of diagnosis, and laboratory data were recorded during that period.

VI (including portal vein, hepatic vein, and inferior vena cava involvement) was assessed in the portal phase of CT and supplemented where appropriate by arterial portography and classified as “present” or “absent.” Response assessments according to mRECIST^20^, ^21^ were made within the 6 to 9 weeks following the first TACE treatment. mRECIST response was categorized as complete response (CR), partial response (PR), SD, and progressive disease (PD). mRECIST data were available in eight of the 17 cohorts (2,688 patients). This analysis did not take into account further TACE treatments undertaken after the first one. Liver function was assessed by the Child‐Pugh grade (as graded by the local investigator) and the albumin‐bilirubin (ALBI) score, the latter being graded according to the published cut‐off points.^24^ Grades 1, 2, and 3 refer to good, intermediate, and poor liver function, respectively. Data on treatment of hepatitis C with direct‐acting antivirals (DAAs) were not collected, but an estimate of the number who might have received this therapy was gained by assessing the date of TACE treatment, assuming there were only a very limited number who would receive DAAs before January 2012.

After generation of the models, as described below, they were externally validated in independent data sets from China and Germany, representing “Eastern” and “Western” cohorts respectively. External validation and calibration were undertaken using methods described by Royston and Altman.^25^, ^26^

### Statistical Methods

Analysis was carried out using Stata/SE 14.1 (StataCorp, TX). Continuous variables were reported as the mean (with standard deviation) or median (with interquartile range), the latter for variables with skewed distributions. Categorical variables were presented as percentages. Logarithmic transformation (log_10_) was applied to skewed variables. Overall survival (OS) was calculated from date of treatment to date of death. Patients who were still alive were censored at date of last follow‐up. Survival curves were plotted using the Kaplan‐Meier (KM) method. For the Post‐TACE‐Predict model, which considers mRECIST response, OS was calculated from the date of response assessment rather than from the date of treatment. Patients with missing data were excluded.

All patients, excluding those from the largest Eastern (Xi’an, n = 786) and Western (Freiburg, n = 407) cohorts, were randomly split into two equally sized groups (n = 1,714), one for deriving the model(s) and one for internal validation of the model (Supporting Fig. S1A). Patients were randomly split by generating a pseudorandom number from a uniform distribution (0, 1) for each patient, followed by shuffling patients by sorting these random numbers. Subsequently, the first half of the patients was labeled as the “training set,” and the second half was labeled as the “internal validation set.” External validation was then conducted using Xi’an and Freiburg data sets. Before construction of the models, the applicability of the original HAP and the subsequent mHAP‐III models^13^, ^15^ was tested on all four subgroups.

The clustering structure of the data set (i.e., the correlation between observations within a center) was taken into account in the statistical analysis. Robust estimates of the standard errors and variance‐covariance matrix were obtained by considering the underlying intracenter correlation (option *vce(cluster clustvar)* in Stata). Multivariable models were built by backward selection of variables significant at the 10% level. The hazard ratio (HR), 95% confidence interval (CI), and *P* values were reported. The proportional hazards assumption of the models was tested by examining the plots of scaled Schoenfeld residuals against time for each variable.

Two multivariable Cox regression models were generated:

- Pre‐TACE‐Predict model: comprising variables available at baseline, before treatment.
- Post‐TACE‐Predict model: incorporating first mRECIST response in addition to baseline features. Not all the cohorts had the mRECIST response recorded; therefore, a smaller set of patients was used (n = 2,688). This set of patients was divided into four subgroups (training, internal, and two external validation samples), as illustrated in Supporting Fig. S1B.

The linear predictor was derived using the coefficients of each model. To generate four risk categories, reported cutoffs were applied to the linear predictor of the training set at its sixteenth, fiftieth, and eighty‐fourth centiles.^25^ The same cutoffs were used for subsequent groupings in the other cohorts. KM survival curves according to the risk categories were plotted for each of the training and validation sets. Median OS (with 95% CIs), HR, and *P* values comparing the HR of the reference group (least risk category) to the others were also reported. Prognostic performance of the models (using the nonstratified linear predictor) was measured by Harrell’s C, Gönen and Heller’s K, and Royston‐Sauerbrei’s $R_{D}^{2}$.^25^, ^27^, ^28^

Models were calibrated by comparing model‐predicted versus observed survival curves. Model‐predicted mean survival curves were generated by applying fractional polynomial regression to approximate the log baseline cumulative hazard function as a smooth function of time.^25^ Model‐predicted versus KM estimates were then plotted according to each risk category in the derivation and validation sets.

## Results

Within the substudy, the HAP score could clearly identify four distinct prognostic subgroups, both in patients undergoing resection and in those receiving sorafenib for advanced HCC (Supporting Fig. S2A,B). The median OS according to each HAP score and the HR and *P* values are shown in Supporting Table S1.

The baseline demographics of the patients from each center are shown in Tables 1 and 2. The percentage of patients who had undergone TACE treatments before January 1, 2012, and January 1, 2013, was 68% and 75.5%, respectively. The percentage of patients with missing data in at least one of the model variables was 14% (training set). For each variable individually, the percentage of missing data was ≤5%.

mRECIST assessments were undertaken within 9 weeks after first TACE for the majority of patients (94.6%) with a mean (standard deviation) of 5.5 weeks (6.8).

The overall median survival for the entire group of patients who underwent TACE was 19.9 months (95% CI: 19.1, 20.7), ranging from 13.7 (95% CI: 9.4, 16.9) to 33.8 (95% CI: 27.4, 39.0). Of all the patients, 2.2% (98/4,486) had more than one cause recorded.

### Application of the HAP and mHAP‐III Scores

The HAP score and the mHAP‐III score were applied to the present data set. The latter score does not categorize patients into risk categories but provides individual‐level prognostication, and this will be compared with HAP later (see the Model Comparisons section). The HAP score stratified the patients into four risk categories in all four subgroups (Supporting Fig. S3A‐D). The median OS according to each HAP score as well as the HR and *P* values are shown in Supporting Table S1.

### Univariable Cox Regressions

The results from the univariable Cox regression analysis based on the training set are shown in Supporting Table S2. Sex, cause, tumor number, tumor size, VI, AFP, and bilirubin were found to be statistically significant prognostic variables. When survival was assessed from date of response assessment (instead of date of treatment), mRECIST response (following first TACE), cause, tumor number, tumor size, VI, AFP, and bilirubin significantly influenced prognosis.

### Multivariable Cox Regressions

#### Pre‐TACE‐Predict

**Figure 1:**
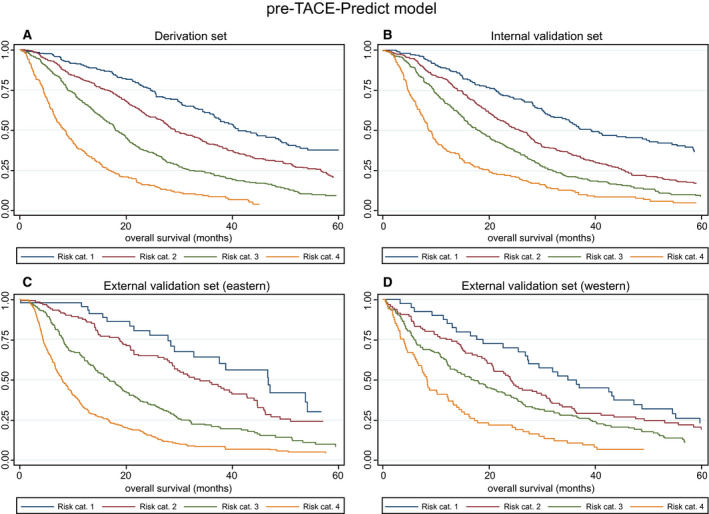
Survival according to risk categories as defined by the Pre‐TACE‐Predict model. KM survival curves in the (A) derivation, (B) internal validation, (C) Eastern external validation, and (D) Western external validation sets. Abbreviation: cat., category.

The model confirmed the prognostic influence of the variables in the mHAP‐III model, namely tumor number, tumor size, AFP, albumin, and bilirubin, in addition to VI and cause (Table 3). It produced four distinct risk categories in each of the four subgroups (Fig. 1A‐D). There was no statistically significant difference between the two lowest risk categories in the external validation sets, probably attributable to the low patient numbers in risk category 1 (n = 40‐44) (Table 4). Median OS ranged from 35 to 47 months in risk category 1 to 8 to 9 months in risk category 4 (Table 4). The formula used to generate the curves in Fig. 1 was as follows:

(1) $$\begin{array}{c} \text{Linear predictor}\;=\;0.313\;\times \;\text{tumor number}\;\left(0\;=\;\text{solitary},\;1\;=\;\;\text{multifocal}\right)+\;\\ 1.252\;\times \;\log_{10}\;\text{tumor size}\;\left(\text{cm}\right)+\;\\ 0.230\;\times \;{\text{baseline log}}_{10}\;\text{AFP}\;\left(\text{ng}/\text{mL}\right)+\\ -\;0.0176\;\times \;\text{baseline albumin}\;\left(g/L\right)+\\ 0.458\;\times \;{\text{baseline log}}_{10}\;\text{bilirubin}\;\left(\mu \text{mol}/L\right)+\;\\ 0.437\;\times \;\text{VI}\;\left(0\;=\;\text{no},\;1\;=\;\text{yes}\right)\\ +0.149\;\times \;\text{HBV}\;(0\;=\;\text{no},\;1\;=\;\text{yes})+\;\\ 0.333\;\times \;\text{alcohol}\;\left(0\;=\;\text{no},\;1\;=\;\text{yes}\right)+\;\\ 0.211\;\times \;\text{other cause if not HCV}/\text{HBV}/\text{alcohol}\;(0\;=\;\text{no},\;1\;=\;\text{yes}) \end{array}$$

**Table 3:** Multivariable Cox Regression Model

Variables	Pre‐TACE‐Predict Model		Post‐TACE‐Predict Model	
	HR (95% CI)	*P* Value	HR (95% CI)	*P* Value
Tumor number				
Solitary	1		1	
Multiple	1.367 (1.146, 1.630)	0.001	1.229 (1.043, 1.450)	0.014
log_10_ Tumor size (cm)	3.497 (2.678, 4.567)	<0.0001	3.091 (1.689, 5.659)	<0.0001
Baseline log_10_ AFP (ng/mL)	1.258 (1.208, 1.311)	<0.0001	1.159 (1.065, 1.261)	0.001
Baseline albumin (g/L)	0.983 (0.966, 0.999)	0.042	N/A	N/A
Baseline log_10_ bilirubin (µmol/L)	1.581 (1.139, 2.194)	0.006	2.118 (1.466, 3.060)	<0.0001
VI				
No	1		1	
Yes	1.549 (1.185, 2.025)	0.001	1.563 (1.004, 2.433)	0.048
Cause				
HCV	1		N/A	N/A
HBV	1.160 (1.030, 1.307)	0.015	N/A	N/A
Alcohol	1.395 (1.049, 1.854)	0.022	N/A	N/A
Other	1.235 (1.017, 1.499)	0.033	N/A	N/A
First mRECIST response				
CR	N/A	N/A	1	
PR	N/A	N/A	1.598 (1.066, 2.396)	0.023
SD	N/A	N/A	3.138 (2.126, 4.630)	<0.0001
PD	N/A	N/A	3.871 (2.553, 5.871)	<0.0001

**Table 4:** Median OS (Months) According to the Risk Categories

Figure	Risk Stratification	Risk Category	N	Median OS (95% CI)	Hazard Ratio (95% CI)	*P* Value
1A Derivation set	Pre‐TACE‐Predict model	1	233	41.02 (36.84, 49.24)	1	
		2	496	29.18 (27.20, 33.49)	1.57 (1.27, 1.95)	<0.0001
		3	495	17.99 (16.81, 19.93)	2.59 (2.10, 3.20)	<0.0001
		4	231	8.36 (6.84, 9.77)	5.44 (4.31, 6.86)	<0.0001
1B Internal validation set	Pre‐TACE‐Predict model	1	255	39.18 (34.44, 51.77)	1	
		2	483	25.89 (23.09, 27.89)	1.58 (1.29, 1.93)	<0.0001
		3	499	18.22 (15.99, 20.23)	2.26 (1.86, 2.75)	<0.0001
		4	219	8.65 (7.73, 9.97)	3.93 (3.15, 4.90)	<0.0001
1C External validation set (Eastern)	Pre‐TACE‐Predict model	1	44	46.68 (29.05, 54.05)	1	
		2	124	33.82 (28.68, 42.66)	1.36 (0.85, 2.19)	0.201
		3	228	16.88 (14.11, 19.34)	2.66 (1.71, 4.15)	<0.0001
		4	330	7.93 (6.94, 9.08)	4.94 (3.19, 7.65)	<0.0001
1D External validation set (Western)	Pre‐TACE‐Predict model	1	40	34.77 (26.81, 47.24)	1	
		2	96	23.95 (19.64, 30.69)	1.33 (0.89, 1.98)	0.165
		3	155	17.11 (12.63, 22.50)	1.74 (1.19, 2.53)	0.004
		4	73	8.29 (6.28, 12.27)	2.99 (1.97, 4.53)	0.0001
2 All patients	mRECIST	CR	625	42.83 (38.83, 46.68)	1	
		PR	745	22.70 (21.09, 24.21)	1.99 (1.71, 2.31)	<0.0001
		SD	765	14.28 (13.03, 15.76)	2.95 (2.56, 3.40)	<0.0001
		PD	496	8.85 (7.87, 10.13)	4.51 (3.87, 5.26)	<0.0001
3A Derivation set	Post‐TACE‐Predict model	1	101	55.53 (47.53, NR)	1	
		2	218	30.26 (26.05, 34.61)	2.50 (1.68, 3.72)	<0.0001
		3	214	17.93 (15.26, 20.46)	5.03 (3.40, 7.42)	<0.0001
		4	92	8.36 (6.88, 9.34)	12.35 (8.06, 18.93)	<0.0001
3B Internal validation set	Post‐TACE‐Predict model	1	106	51.18 (37.37, 78.22)	1	
		2	221	27.50 (24.97, 35.76)	2.14 (1.48, 3.08)	<0.0001
		3	220	19.47 (16.51, 24.21)	3.37 (2.36, 4.80)	<0.0001
		4	79	8.09 (5.72, 10.53)	7.55 (5.01, 11.39)	<0.0001
3C External validation set (Eastern)	Post‐TACE‐Predict model	1	38	49.80 (28.06, 70.03)	1	
		2	99	31.22 (27.53, 37.53)	1.72 (1.02, 2.90)	0.043
		3	203	21.18 (17.60, 24.97)	2.39 (1.46, 3.92)	0.001
		4	375	7.01 (6.09, 7.80)	5.94 (3.68, 9.59)	<0.0001
3D External validation set (Western)	Post‐TACE‐Predict model	1	9	25.13 (11.68, NR)	1	
		2	41	34.31 (23.39, 47.11)	1.44 (0.57, 3.67)	0.444
		3	147	22.96 (18.78, 27.34)	1.81 (0.74, 4.44)	0.192
		4	144	9.84 (6.35, 11.78)	3.50 (1.43, 8.56)	0.006

where HCV is the reference group for cause.

To generate the four risk categories, the following cutoffs were applied: ≤0.94 (risk category 1), >0.94 to ≤1.47 (risk category 2), >1.47 to ≤2.10 (risk category 3), and >2.10 (risk category 4).

To calculate the probability of survival at *t* months for a given patient, the following equation was used:

(2) $$S\left(t\right)=S_{0}\left(t\right)\;\exp\left(xb-1.47\right)$$

where *S*_0_(*t*) is 0.89, 0.74, 0.48, and 0.32 for probability at 6, 12, 24, and 36 months, respectively.

#### Post‐TACE‐Predict Model

**Figure 2:**
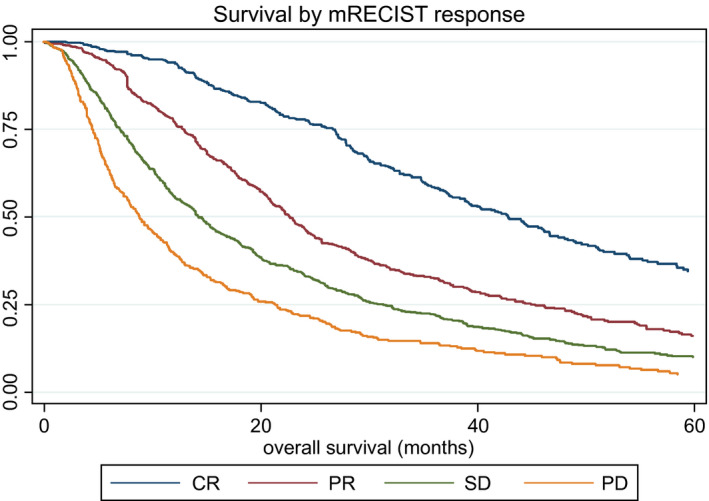
KM survival curves according to mRECIST response.

Response, as assessed by mRECIST, clearly impacted median survival, which ranged from 42.83 months (95% CI: 38.83, 46.68) in those achieving CR to 8.85 months (95% CI: 7.87, 10.13) in those with PD (Fig. 2), although these figures should be treated with caution because the different response cohorts had different baseline features that would also influence survival. Nonetheless, in the Post‐TACE‐Predict model, response was clearly an independent prognostic factor (Table 3), in addition to tumor number, tumor size, AFP, bilirubin, and VI.

**Figure 3:**
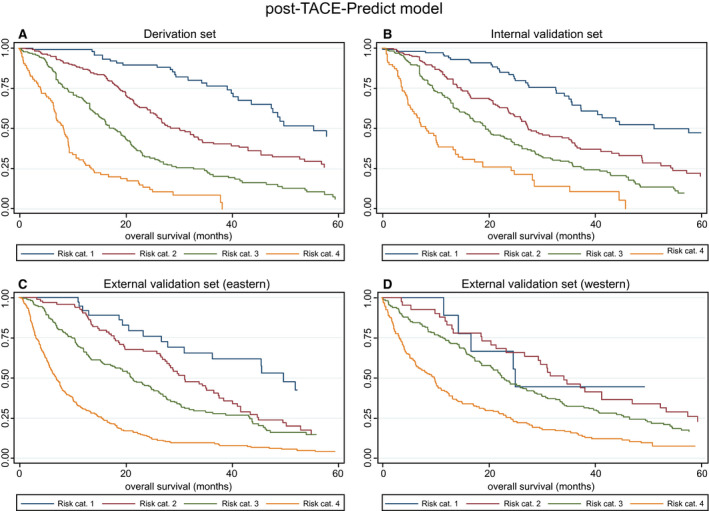
Survival according to risk categories as defined by the Post‐TACE‐Predict model. KM survival curves in the (A) derivation, (B) internal validation, (C) Eastern external validation, and (D) Western external validation sets. Abbreviation: cat., category.

Four distinct risk categories were observed in each of the four subgroups (Fig. 3A‐D); however, there was some overlap between the two lowest risk categories in the Western external validation set, in which the patient numbers were again very low, with only 9 patients in risk category 1. The median OS of the risk categories ranged from 25 to 56 months in risk category 1 to 7 to 10 in risk category 4 (Table 4). The formula to generate the curves in Fig. 3 was as follows:

(3) $$\begin{array}{c} \text{Linear predictor}\;=\;0.207\;\times \;\text{tumor number}\;\left(0\;=\;\text{solitary},\;1\;=\;\text{multifocal}\right)\;+\;\\ 1.129\;\times \;\log_{10}\;\text{tumor size}\;\left(\text{cm}\right)+\;\\ 0.147\;\times \;{\text{baseline log}}_{10}\;\text{AFP}\;\left(\text{ng}/\text{mL}\right)+\;\\ 0.750\;\times \;{\text{baseline log}}_{10}\;\text{bilirubin}\;\left(\mu \text{mol}/L\right)+\;\\ 0.447\;\times \;\text{VI}\;\left(0\;=\;\text{no},\;1\;=\;\text{yes}\right)+\\ 0.469\;\times \;\text{PR}\;\left(0\;=\;\text{no},\;1\;=\;\text{yes}\right)+\\ \;1.143\;\times \;\text{SD}\;\left(0\;=\;\text{no},\;1\;=\;\text{yes}\right)+\;\\ 1.354\;\times \;\text{PD}\;(0\;=\;\text{no},\;1\;=\;\text{yes}) \end{array}$$

where CR is the reference group for mRECIST.

To generate the four risk categories, the following cutoffs were applied (as determined by the sixteenth, fiftieth, and eighty‐fourth centiles): ≤1.82 (risk category 1), >1.82 to ≤2.49 (risk category 2), >2.49 to ≤3.37 (risk category 3), and >3.37 (risk category 4).

To calculate the probability of survival at *t* months for a given patient, the following equation was used:

(4) $$S\left(t\right)=S_{0}\left(t\right)\;\exp\left(xb-2.49\right)$$

where *S*_0_(*t*) is 0.92, 0.79, 0.52, and 0.36 for probability at 6, 12, 24, and 36 months, respectively.

For routine clinical application, a simple online calculator (based on (1), (2), (3), (4)) that takes the variables from the model(s) and returns the scores, the risk category, and survival likelihood at six monthly intervals between 6 and 36 months after TACE for the individual patient was developed and is available at https://jscalc.io/calc/2omTfeWrmOLc41ei.

### Model Calibration

Plots of KM estimates versus pre‐TACE‐predicted and post‐TACE‐predicted survival curves were, overall, very similar (Supporting Figs. S4 and S5A‐D), although it should be noted that there was an overlap in the CIs for the KM estimates in the lowest two risk categories of the external validation sets. This was reflected by the non–statistically significant HRs, as stated above; low patient numbers may have contributed to this.

### Model Comparisons

Table 5 summarizes the comparisons between the different models by Harrell’s C, Gönen and Heller’s K, and Royston‐Sauerbrei’s $R_{D}^{2}$. It confirms that mHAP‐III performs better than the HAP score. It also shows a trend of increasingly better survival prediction performance from mHAP‐III to the pre‐TACE and then post‐TACE models.

**Table 5:** Model Performance

Goodness of Fit Test	Data Set	HAP (SE)	mHAP‐III (SE)	Pre‐TACE‐Predict Model (SE)	Post‐TACE‐Predict Model (SE)
Harrell’s C index	Training	0.616 (0.010)	0.651 (0.011)	0.682 (0.010)	0.723 (0.013)
	Internal validation	0.624 (0.009)	0.649 (0.010)	0.659 (0.010)	0.693 (0.016)
	External validation (Eastern)	0.640 (0.012)	0.687 (0.012)	0.707 (0.012)	0.730 (0.011)
	External validation (Western)	0.597 (0.015)	0.618 (0.016)	0.613 (0.017)	0.631 (0.017)
Gönen & Heller’s K	Training	0.592 (0.010)	0.633 (0.010)	0.651 (0.010)	0.680 (0.012)
	Internal validation	0.598 (0.010)	0.617 (0.010)	0.623 (0.010)	0.654 (0.013)
	External validation (Eastern)	0.605 (0.013)	0.655 (0.011)	0.667 (0.012)	0.681 (0.012)
	External validation (Western)	0.581 (0.014)	0.545 (0.023)	0.587 (0.016)	0.596 (0.016)
Royston‐Sauerbrei’s $R_{D}^{2}$	Training	0.078 (0.015)	0.132 (0.021)	0.181 (0.020)	0.262 (0.034)
	Internal validation	0.087 (0.016)	0.111 (0.020)	0.120 (0.020)	0.185 (0.030)
	External validation (Eastern)	0.096 (0.023)	0.184 (0.024)	0.209 (0.028)	0.243 (0.034)
	External validation (Western)	0.059 (0.023)	0.050 (0.019)	0.058 (0.022)	0.073 (0.026)

SEs were estimated from 200 bootstrap samples.

Abbreviation: SE, standard error.

## Discussion

These models, based on TACE response, stratify survival better than the currently available HAP and mHAP‐III models. The median OS was 19.9 months, almost identical to the figures of 19.4 months reported by Lencioni in a large systematic review of published trials involving TACE between 1980 and 2013.^29^ This suggests that this cohort is representative of the current international practice of TACE for HCC. Furthermore, the clear demonstration that the degree of response has a major and independent impact on survival strongly supports the contention that TACE is indeed altering the natural history.^29^

The heterogeneity of intermediate‐stage HCC and the widespread use of TACE outside recommended guidelines has encouraged the development of scores that can predict survival after TACE using baseline clinical features.^10^, ^12^, ^14^, ^30^, ^31^, ^32^ The first of these, the HAP score, has been internationally validated and enhanced by the addition of a fifth variable, namely tumor number.^13^, ^23^, ^33^ Recognizing the limitations of points‐based scores, Cappelli et al. built a model (known as mHAP‐III) based on the mHAP‐II score but using the same variables in their continuous form, which permitted individual patient prognostication.^15^ Sposito et al. subsequently validated the mHAP‐III model in an independent data set of 298 patients and confirmed its superiority to both HAP and mHAP‐II.^34^ The reported STATE and START scores^8^ also appear to be valuable in identifying patients as poor or good candidates for TACE but require variables such as C‐reactive protein, which were not routinely measured in the centers involved in the present study. Similarly, the ABCR score^35^ that combines four variables (AFP, BCLC stage, change in Child‐Pugh score, and tumor response) aims to identify those with poor prognosis who may not achieve benefit from further TACE. Again, the variables were not available to make a direct comparison (particularly the actual CP scores), but in the follow‐up prospective study, an attempt will be made to collect the requisite variables to permit comparison of STATE, START, and ABCR with the current models. It will also be possible to investigate other and potentially valuable additional variables, such as performance status and presence or absence of cirrhosis. Nonetheless, the additional significant variables, the individual patient prognostication, and the extensive international validation are likely to represent a real improvement on existing scores.

The online calculator (TACE‐Predict) provides a simple utility for individual patient‐level prognostication. It also permits easy graphical assessment of the importance of the various prognostic variables on ultimate survival. The model involves readily available, routinely recorded clinical variables. The clear correlation of survival with degree of response (as assessed by mRECIST) is consistent with past findings.^36^ Using these calculators, clinicians will be able to predict the probability of survival at the individual patient level, thereby furthering the ultimate aim of matching “personalized prognosis” to “personalized therapy.” For example, either before proposed first TACE or at the time of first response assessment, the clinician will be able to consider if the predicted survival is appropriate in the light of the potential side effects and toxicities of TACE. This may be particularly clinically valuable in the situation where the predicted outcome is poor, and consideration might be given to systemic therapy. Moreover, all the models were validated on large cohorts of patients to demonstrate the applicability of this approach to both the Eastern and Western practice.

It is acknowledged that the TACE procedure is unlikely to be entirely consistent across centers. However, this limitation applies equally to all TACE studies, including those on which current guidelines are based. Similarly, there must be interobserver variation in mRECIST classification. Although such variation may be overcome in the clinical trial setting by centralized review of relevant scans, this cannot be a solution in clinical practice. Hence, we made the pragmatic decision that mRECIST classification, as assessed by the local investigator, would be used in the present study.

Nonetheless, there is considerable heterogeneity in achievement, for example, of CR. The most likely explanation is that those centers with the highest CR (Italy and Egypt) had smaller tumors, more early‐stage disease, less VI, and more solitary nodules. The very clear separation of survival according to mRECIST (Fig. 2) suggests that a valid parameter is indeed being measured. It is recognized that calculating OS from mRECIST assessment introduces a degree of variability into the post‐TACE model because of the differing times of imaging between patients. This source of variability is, however, intrinsic to the time at which mRECIST is assessed, which is patient‐specific, and would affect any model that includes mRECIST, regardless of whether OS is calculated in the model from date of mRECIST response or date of treatment.

The inherent limitations of a retrospective study are also acknowledged. First, there are several other baseline features that are likely to impact OS and could be included in the analysis, specifically, the extent of VI^11^ (as opposed to a simple binary classification of present or absent), the structure of the tumor (pseudocapsule versus infiltrative), or liver function kinetics. However, such parameters are not routinely collected, and their inclusion in the study would have limited the applicability of the models. Second, only the first TACE in this study was considered. Assessment of the response after the second TACE or using the “best response” are also options, but both would limit the applicability of the model. Furthermore, patients were excluded who had received TACE as a “bridge to transplantation.” An alternative approach would have been to recruit such patients and censor at the time of transplantation, but, given the usually short period of time between TACE and transplantation, this alternative approach would only have minimal impact on the models. In the prospective study, the investigation of the impact of all the above limitations will be feasible.

As in many areas of hepatology, the recent availability of curative therapies for HCV will have a broad impact on predictive and therapeutic studies. At present, it is not known whether patients who have developed HCC after a DAA‐induced sustained virological response should be classified as HCV‐positive in the models, but the number of such cases is likely to be relatively small. The great majority of patients in the present study were recruited before DAAs became widely available. The question of how to assign cause as a variable remains challenging, even in a prospective study. Although cause was shown to be an important prognostic factor, with patients who were HCV‐positive surviving longer, several of the cases had multiple causes; however, even with a large data set of more than 4,000 cases, the numbers in individual subgroups, such as those with HCV and alcohol excess or both HBV and HCV, remain too small for meaningful statistical analysis. NAFLD is an increasingly important causal factor in HCC development; however, there are no internationally agreed‐on criteria for diagnosis of NAFLD in the setting of HCC. Furthermore, it is acknowledged that the diagnosis of NAFLD is difficult in the setting of cirrhosis (which is the case in most HCCs) because the characteristic features of NAFLD have often “burned out” and are unrecognizable by the time consequential cirrhosis has developed. For all these reasons, it is concluded that the fairest statement of cause is, as used here, simply HBV or HCV or “other.”

Many programs offer TACE with DEB‐TACE as opposed to conventional TACE. This has the advantage of offering a better pharmacokinetic profile by means of sustained and controlled drug release.^37^ Published meta‐analyses, however, suggest that there is little difference in terms of impact on outcome,^38^, ^39^, ^40^, ^41^, ^42^ albeit with a decreased need for repeat sessions.^43^ This was therefore not included in the analysis.

International guidance and expert reviews quote overall post‐TACE survival of more than 30 months.^1^ If the analysis of the data set is confined to those that strictly align with TACE guidelines, survival is indeed in the order of 30 months, and in the model, just using baseline features, some subgroups surviving more than 40 months are identified. The overall median survival of 19.9 months is also similar to that reported in a recent review,^29^ suggesting that TACE is often prescribed for patients beyond BCLC B. The model and online calculator can help rationalize the use of TACE and avoid interventions with an expected poor prognosis and the associated risks.

In summary, an extensively validated and TACE‐specific model based on routinely available clinical features and response after first TACE is presented. The model and its associated online calculator permit patient‐level prognostication and may help clinicians rationalize the use of TACE by avoiding intervention in patients with a predicted poor prognosis.
