# Supplementary material for: Prediction of Survival Among Patients Receiving Transarterial Chemoembolization for Hepatocellular Carcinoma: A Response‐Based Approach
Source: Hepatology. 2020 May 27;72(1):198–212. doi: 10.1002/hep.31022 (PMC7496334; doi:10.1002/hep.31022)

Supplementary figure legends

Supplementary figure 1: Patient number breakdown into training and validation sets using (A) overall patients and (B) patients with mRECIST response.

Supplementary figure 2: Kaplan-Meier survival curves according to the HAP score in patients undergoing (A) resection and (B) sorafenib treatment.

Supplementary figure 3: Kaplan-Meier survival curves according to the HAP score in the (A) derivation, (B) internal validation, (C) eastern external validation and (D) western external validation sets.

Supplementary figure 4: Plots comparing Kaplan-Meier estimates with pre-TACE model predicted mean survival curves in the (A) derivation, (B) internal validation, (C) eastern external validation and (D) western external validation sets.

Supplementary figure 5: Plots comparing Kaplan-Meier estimates with post-TACE model predicted mean survival curves in the (A) derivation, (B) internal validation, (C) eastern external validation and (D) western external validation sets.

Supplementary tables

Supplementary table 1: Median overall survival (months) according the risk categories

Supplementary figure	Risk stratification	Risk category	N	Median OS (95% CI)	Hazard ratio (95% CI)	p-value
2A<br>Patients undergoing surgical resection	HAP score	A	1430	110.16 (95.51, 127.07)	1	
		B	1333	73.91 (61.70, 82.20)	1.73 (1.51, 1.97)	<0.0001
		C	635	36.88 (29.52, 43.36)	2.93 (2.52, 3.41)	<0.0001
		D	158	16.99 (12.00, 24.11)	4.54 (3.62, 5.69)	<0.0001
2B<br>Patients with advanced HCC receiving sorafenib	HAP score	A	178	16.45 (14.54, 20.76)	1	
		B	313	11.84 (10.30, 13.52)	1.42 (1.12, 1.79)	0.003
		C	311	7.17 (6.28, 8.06)	2.30 (1.83, 2.89)	<0.0001
		D	165	4.24 (3.72, 4.70)	4.32 (3.36, 5.56)	<0.0001
4A<br>Derivation set	HAP score	A	280	32.80 (27.30, 40.30)	1	
		B	522	27.37 (23.09, 29.61)	1.28 (1.06, 1.54)	0.009
		C	543	18.72 (16.88, 20.66)	1.81 (1.51, 2.17)	<0.0001
		D	169	9.01 (7.47, 11.71)	3.15 (2.50, 3.96)	<0.0001
4B<br>Internal validation set	HAP score	A	271	33.19 (28.62, 37.20)	1	
		B	551	26.07 (23.09, 28.22)	1.36 (1.13, 1.64)	0.001
		C	489	17.27 (15.33, 19.90)	1.87 (1.55, 2.26)	<0.0001
		D	209	9.34 (7.89, 11.02)	3.50 (2.81, 4.35)	<0.0001
4C<br>External validation set (eastern)	HAP score	A	64	32.80 (29.01, 38.72)	1	
		B	204	21.41 (18.55, 28.26)	1.56 (1.09, 2.23)	0.016
		C	255	12.93 (11.18, 16.51)	2.21 (1.55, 3.15)	<0.0001
		D	204	7.30 (6.12, 8.36)	3.61 (2.52, 5.15)	<0.0001
4D<br>External validation set (western)	HAP score	A	72	30.39 (22.99, 39.41)	1	
		B	113	22.50 (17.11, 26.71)	1.40 (1.02, 1.93)	0.038
		C	117	16.28 (11.35, 20.36)	1.68 (1.22, 2.30)	0.001
		D	63	8.68 (5.92, 13.85)	2.52 (1.76, 3.62)	<0.0001

CI, confidence intervals; HAP, Hepatoma Arterial Prognostic; HCC, hepatocellular carcinoma; OS, overall survival

**Supplementary table 2:** Univariable Cox regression analysis				
**Variables**	**Time starting from date of treatment**		**Time starting from date of response assessment**	
	**Hazard ratio (95% CI)**	**p-value**	**Hazard ratio (95% CI)**	**p-value**
**Age (years)**	0.994 (0.981, 1.006)	0.326	0.997 (0.980, 1.014)	0.725
**Sex**				
Female	1		1	
Male	1.154 (1.059, 1.258)	0.001	0.966 (0.872, 1.071)	0.512
**Aetiology**				
HCV	1		1	
HBV	1.462 (1.210, 1.767)	<0.0001	1.052 (0.627, 1.765)	0.847
Alcoholic	1.473 (1.187, 1.827)	<0.0001	1.340 (1.025, 1.752)	0.032
Other	1.547 (1.216, 1.969)	<0.0001	1.202 (0.970, 1.489)	0.093
**Tumour number**				
Solitary	1		1	
Multiple	1.348 (1.131, 1.606)	0.001	1.536 (1.274, 1.853)	<0.0001
**$\log_{10}$ Tumour size (cm)**	4.082 (3.160, 5.274)	<0.0001	3.775 (2.355, 6.052)	<0.0001
**Vascular invasion**				
No	1		1	
Yes	2.379 (1.858, 3.046)	<0.0001	2.300 (1.194, 4.427)	0.013
**Baseline $\log_{10}$ AFP (ng/ml)**	1.344 (1.267, 1.425)	<0.0001	1.232 (1.170, 1.298)	<0.0001
**Baseline albumin (g/l)**	0.987 (0.968, 1.006)	0.187	1.000 (0.988, 1.011)	0.978
**Baseline $\log_{10}$ bilirubin (μmol/l)**	1.462 (1.021, 2.093)	0.038	2.211 (1.085, 4.506)	0.029
**First mRECIST response**				
Complete response	N/A	N/A	1	
Partial response	N/A	N/A	1.991 (1.377, 2.879)	<0.0001
Stable disease	N/A	N/A	3.801 (2.816, 5.131)	<0.0001
Progressive disease	N/A	N/A	4.386 (3.070, 6.266)	<0.0001

AFP, alpha-fetoprotein; CI, confidence intervals; HBV, hepatitis B virus; HCV, hepatitis C virus; mRECST, modified Response Evaluation Criteria In Solid Tumors

Supplementary Figure 1

A

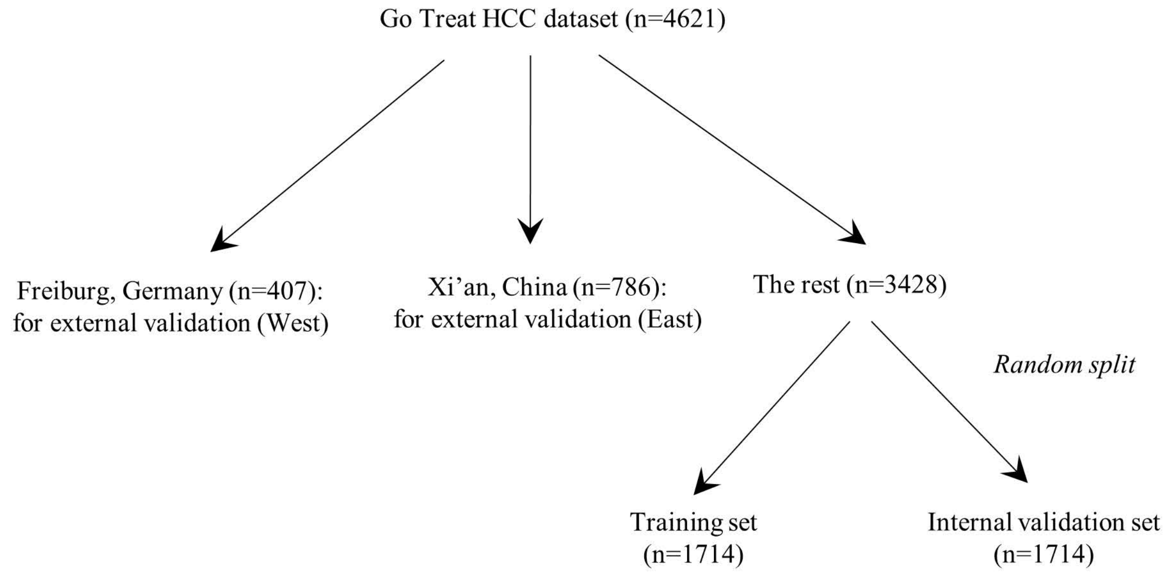

B

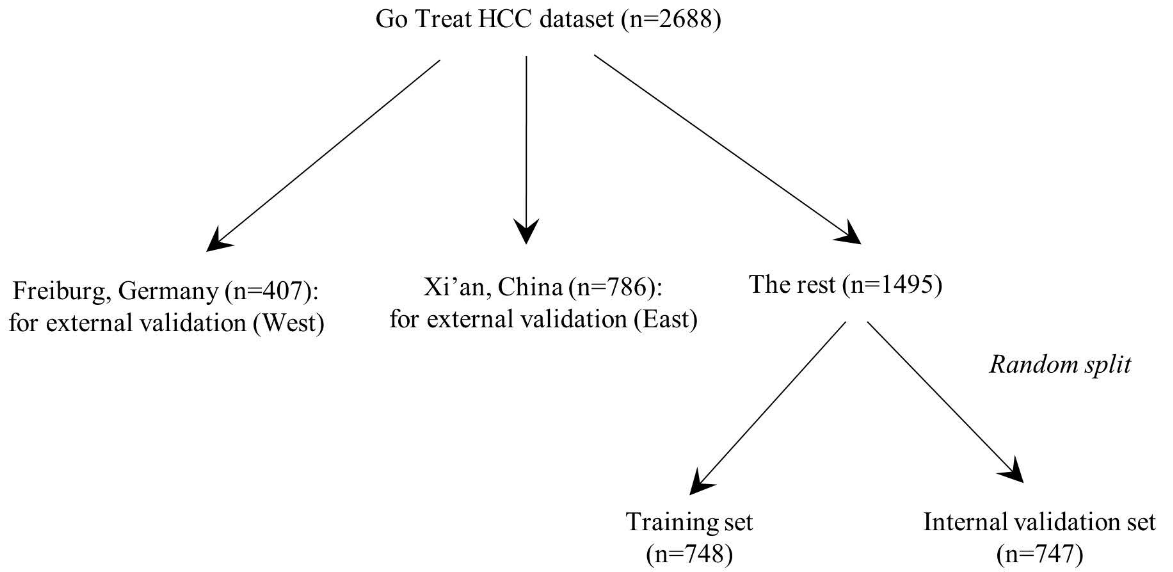

Supplementary Figure 2

HAP score

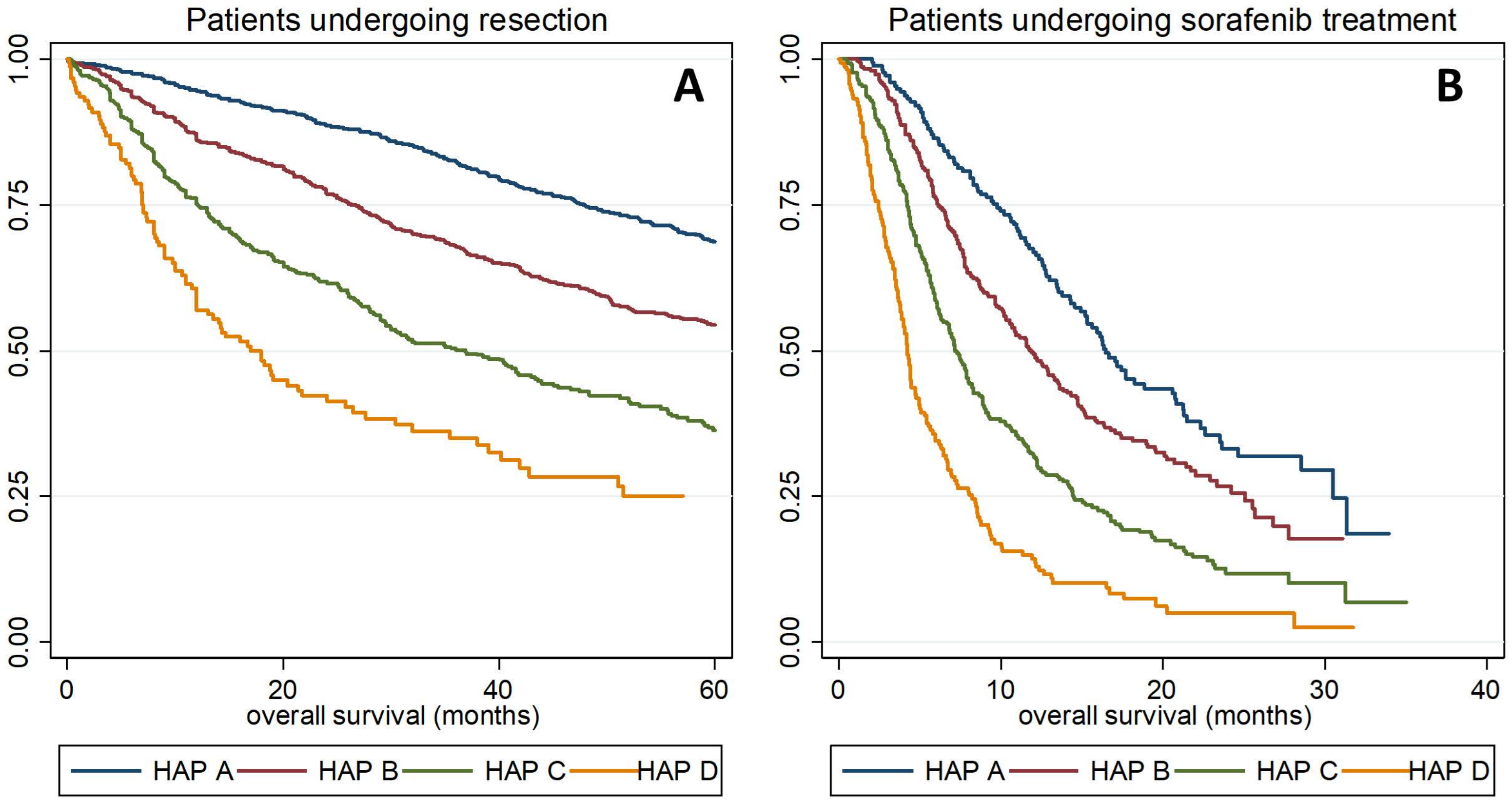

Supplementary Figure 3

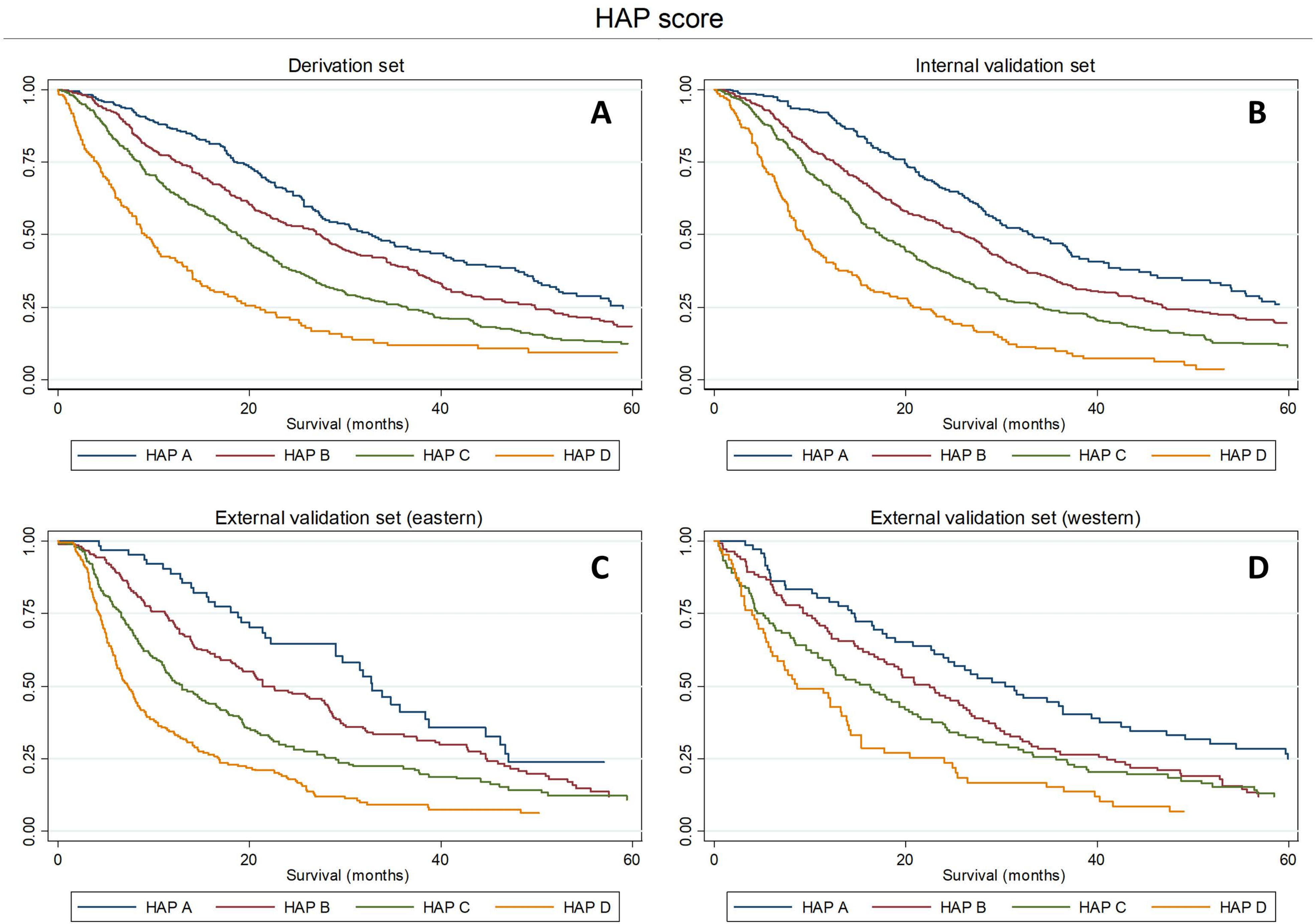

Supplementary Figure 4

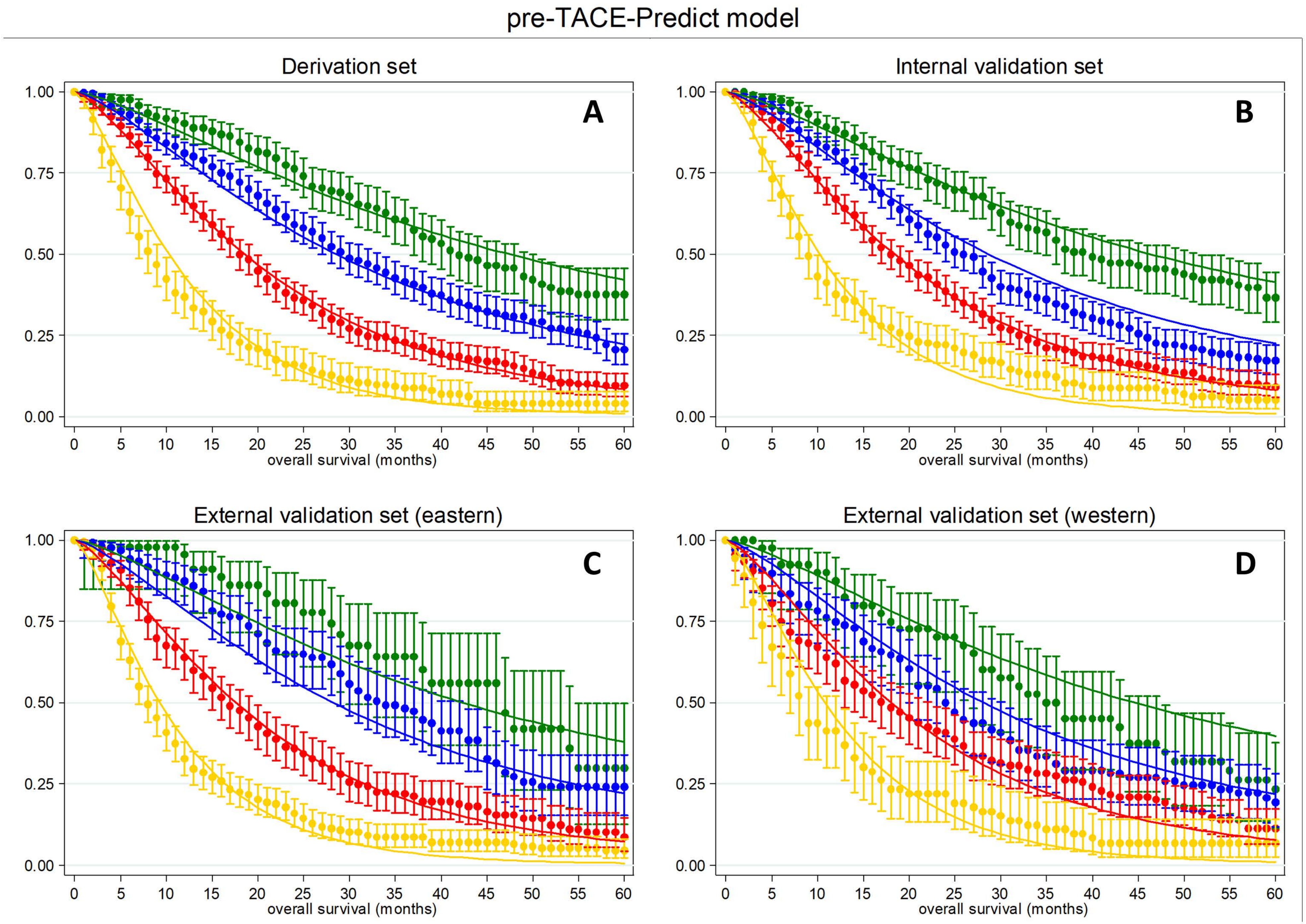

Supplementary Figure 5

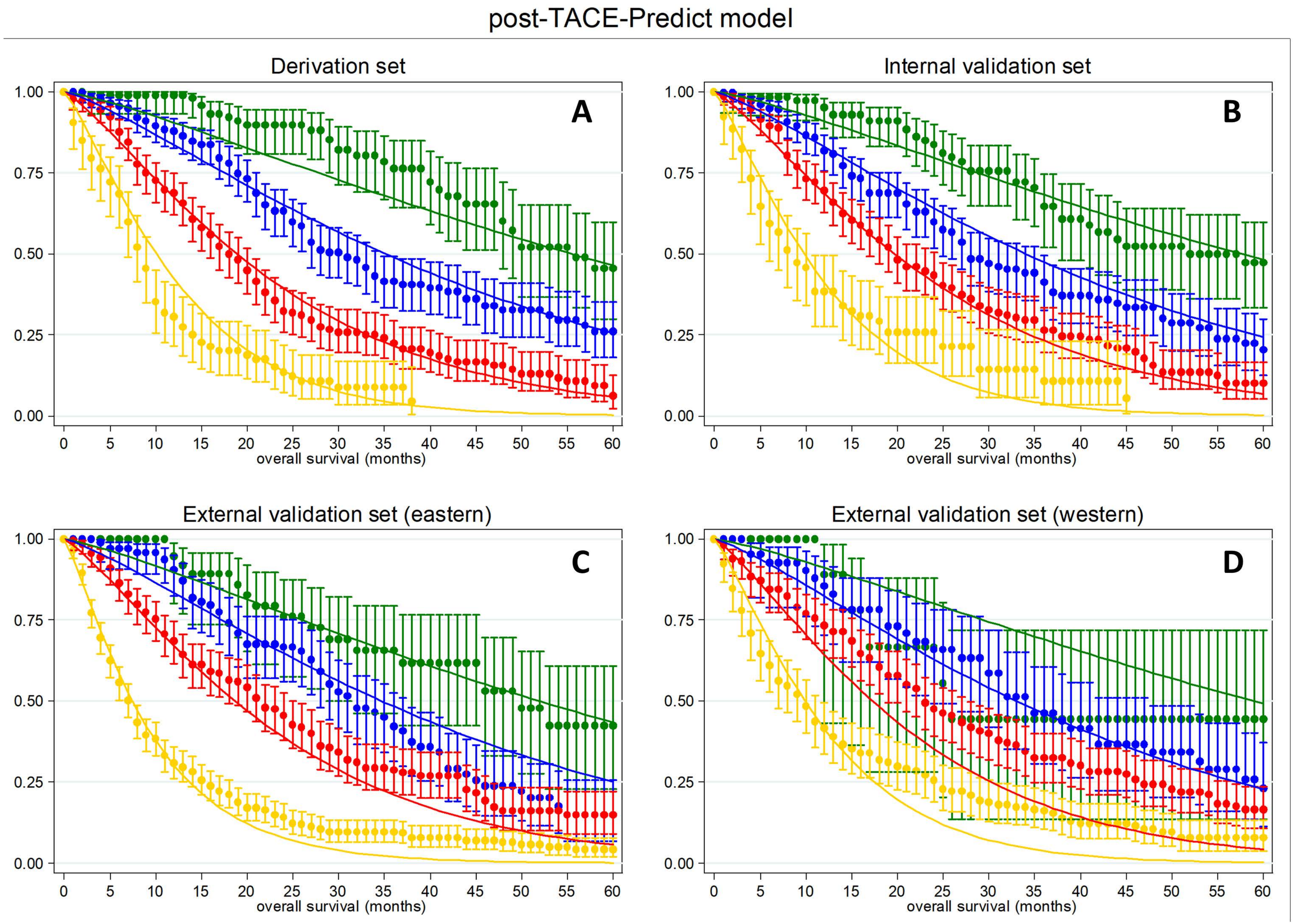

Supplement: Supplementary file 1 [file HEP-72-198-s001.pdf]
